# Sewage-and fertilizer-derived nutrients alter the intensity, diversity, and toxicity of harmful cyanobacterial blooms in eutrophic lakes

**DOI:** 10.3389/fmicb.2024.1464686

**Published:** 2024-11-06

**Authors:** Christopher J. Gobler, Ruth W. Drinkwater, Alexander Anthony, Jennifer A. Goleski, Ann Marie E. Famularo-Pecora, Marcella Kretz Wallace, Nora R. W. Straquadine, Ronojoy Hem

**Affiliations:** School of Marine and Atmospheric Sciences, Stony Brook University, Southampton, NY, United States

**Keywords:** Microcystis, microcystin, harmful algal bloom, harmful algae, fertilizer, sewage, wastewater, wastewater treatment

## Abstract

Cyanobacterial harmful algal blooms (CHABs) are promoted by excessive nutrient loading and, while fertilizers and sewage are the most prevalent external nutrient sources in most watersheds, the differential effects of these nutrient sources on CHABs are unknown. Here, we tracked CHABs and performed experiments in five distinct lakes across the Northern US including Lake Erie. Fertilizers with ammonium and orthophosphate, membrane (0.2 μm)-filtered sewage (dominated by reduced forms of nitrogen) sand-and membrane-filtered sewage (dominated by nitrate), and an inorganic nutrient solution of ammonium and orthophosphate were used as experimental nutrient sources for CHABs at N-equivalent, environmentally realistic concentrations. Phytoplankton communities were evaluated fluorometrically, microscopically, and via high throughput sequencing of the 16S rRNA gene, and levels of microcystin and the δ^15^N content of particulate organic nitrogen (δPO^15^N) were quantified. Fertilizer and both sources of wastewater increased the abundance of cyanobacteria in all experiments across all five lakes (*p* < 0.05 for all) whereas effects on eukaryotic phytoplankton were limited. Sand-filtered sewage contained less P, organic matter, and ammonium but more nitrate and had a 25% less potent stimulatory effect on cyanobacteria than membrane-filtered sewage, suggesting nitrification may play a role in reducing CHABs. Fertilizer increased microcystin levels and decreased the δPO^15^N whereas wastewater increased δPO^15^N (*p* < 0.05 for all). *Microcystis* was the genus most consistently promoted by nutrient sources (*p* < 0.05 in all experiments), followed by *Cyanobium* (*p* < 0.05 in 50% of experiments), with increases in *Microcystis* biomass consistently elicited by membrane-filtered wastewater. Collectively, results demonstrate that differing types of sewage discharge and fertilizers can promote CHAB intensity and toxicity, while concurrently altering CHAB diversity and δPO^15^N. While membrane-filtered sewage consistently favored *Microcystis*, the discharge of sewage through sands muted bloom intensity suggesting sand-beds may represent a tool to remove key nutrients and partially mitigate CHABs.

## Introduction

Blooms of toxic cyanobacteria are an expanding global phenomenon that threaten public health, ecosystems, and economies (Watson et al., 2016; Bullerjahn et al., 2016; Huisman et al., 2018) and excessive nutrient loading is the process most commonly cited as the environmental factor promoting CHABs (Schindler et al., 2008, 2016; O’Neil et al., 2012; Paerl, 2018). Historically, phosphorus (P) was considered the singular nutrient responsible for promoting CHABs (Smith, 1979; Schindler, 1977; Schindler et al., 2008, 2016). A wealth of evidence has emerged during the past two decades, however, establishing that nitrogen (N) loading can also control the growth and/or toxicity of some CHABs (Chaffin et al., 2013; Gobler et al., 2016; Hellweger et al., 2022; Kramer and Gobler, 2023; Kramer et al., 2024).

Globally, anthropogenic sources of nutrients comprise the dominant external nutrient loads in most developed nations (Canfield et al., 2010; Lee et al., 2016). While fertilizer is the largest source of N and P within many watersheds across the planet (Canfield et al., 2010; Fink et al., 2018), within urbanized regions such as the Northeast US and other regions near urban centers, wastewater can be a more important source of nutrients (Castro et al., 2003; Latimer and Charpentier, 2010; Jenny et al., 2016) While reducing nutrients is generally the strategy viewed as the most efficacious approach for mitigating CHABs (Paerl et al., 2016), little is known regarding the differential responses of CHABs to differing nutrient sources. While both synthetic fertilizers and wastewater are generally enriched in N and P, wastewater is further enriched in organic matter that may promote the growth of cyanobacteria (Berman and Chava, 1999; Harke and Gobler, 2013) and/or co-occurring heterotrophic bacterial communities (Paerl, 1977; Kirchman, 2010) and thereby potentially influence CHABs (Jankowiak and Gobler, 2020; Cook et al., 2020; Smith et al., 2021). While wastewater is often directly discharged into surface waters, in some cases, is may be discharged to ground and may ultimately enter surface waters as it flows through a riparian zone (Freeze and Cherry, 1979).

Historically, there have been many instances of CHABs linked to specific nutrient sources. In Lake Erie, North America, the intensification of CHABs, mitigation of CHABs, and re-intensification of CHABs this century have been associated with increasing wastewater discharge in the 1960s, sewage treatment and nutrient mitigation in the 1970s, and an intensification of fertilizer loading this century, respectively (Stumpf et al., 2012; Ho et al., 2017; Reutter, 2019). In China, blooms in Lake Taihu have been linked to increasing nutrient loads from fertilizer and wastewater from the surrounding watershed (Wang et al., 2009, 2019). In Florida, United States, CHABs are common in Lake Okachobee where fertilizers are the dominant source of N and P (He et al., 2014; Ma et al., 2020), whereas downstream intensification of these events has been linked to onsite wastewater flow (Lapointe et al., 2017). Still, to our knowledge, no study to date has directly evaluated the effects of nutrients from fertilizer and differing sources of sewage on CHAB communities.

This study, therefore, was conducted to track CHABs in five lakes across the eastern US, including Lake Erie, and to perform experiments utilizing different nutrient sources (fertilizers, membrane-and sand-filtered and membrane-filtered wastewater as nutrient sources) that were normalized to N levels but differed in their organic carbon and, to a lesser extent, their P content. Phytoplankton communities were evaluated fluorometrically, microscopically, and via high throughput sequencing of the 16S rRNA gene, and levels of microcystin and the δ^15^N content of particulate organic nitrogen (δPO^15^N) were quantified. We hypothesized that all nutrient sources would equally promote phytoplankton communities, preserving initial diversity across cyanobacteria and eukaryotic algae and community structure among cyanobacterial populations.

## Methods

### Study sites and initial sample processing

**Figure 1:**
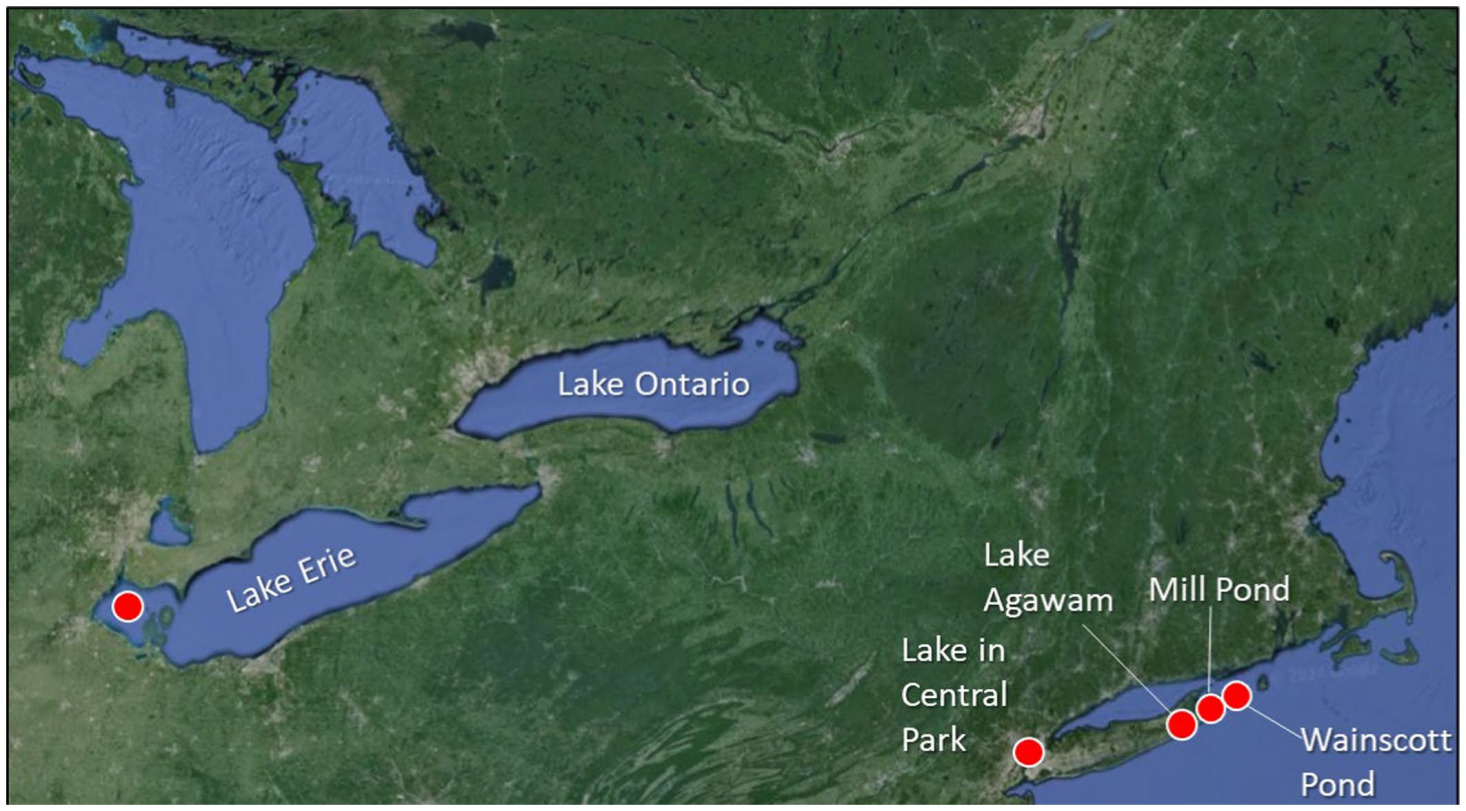
Sampling locations across North America including Lake Erie, the Lake in Central Park, New York City, NY, Lake Agawam in Southampton, NY, Mill Pond in Watermill, NY, and Wainscott Pond in Wainscott, NY. Precise latitude and longitude of each location appears in the methods.

Water was collected from January through December of 2021 from five North American lakes including Lake Agawam, Southampton, NY (40.88148, −72.39256; Figure 1), the Lake in Central Park, New York City, NY (40.77458, −73.97073; Figure 1), Mill Pond, Watermill, NY (40.915906, −72.353753; Figure 1), Wainscott Pond, Wainscott, NY, (40.9282, 72.2416; Figure 1), and Maumee Bay within the western basin of Lake Erie (41.823817, −83.331580; Figure 1), all eutrophic systems, prone to CHABs (Davis et al., 2010; Jankowiak and Gobler, 2020; Jankowiak et al., 2019; Flanzenbaum et al., 2022; Kramer et al., 2024). While Lake Agawam, the Lake in Central Park, Mill Pond, and Wainscott Pond are all small (0.2–0.5 km^2^), shallow (2–4 m), and well-mixed systems, the western basin of Lake Erie is deeper (~11 m maximum depth) and becomes seasonally stratified (Watson et al., 2016). The Lake in Central Park, Mill Pond, Lake Agawam, and Wainscott Pond were sampled weekly-to-monthly during this study while Lake Erie was sampled for several days in August 2001 during a *Microcystis* bloom. On site, surface temperature, dissolved oxygen, and pH were measured with a YSI 556 ProQuatro multiparameter sonde and a 10% HCl-washed, 20-L polycarbonate carboy was collected and transported to the Stony Brook – Southampton Marine Science Center or the Ohio State Stone Laboratory on Lake Erie. *In vitro* chlorophyll-*a* concentrations of planktonic diatoms, chlorophytes, and cyanobacteria were measured using a bbe Moldaenke Fluoroprobe, yielding fluorescence-based biomass estimates of each group expressed in μg chlorophyll *a* L^−1^ (Beutler et al., 2002; Jankowiak et al., 2019). Duplicate samples were collected for the analysis of whole water (dissolved and particulate) microcystin. Toxin samples were analyzed by an ABRAXIS® Microcystin/Nodularians test kit according to the manufacturer’s (Gold Standard Diagnostics) procedures which included a 3X freeze/thaw cycle.

### Nutrient amendment experiments

Over the sampling year, nutrient amendment experiments were performed twice for Lake Agawam, three times for Mill Pond, once for the Lake in Central Park, once for Wainscott Pond, and once for Lake Erie (Table 1). Water was collected in triplicate, 20 L polyethylene carboys and transported to Stony Brook University’s Marine Sciences Center in Southampton, NY. Water was distributed into 2.75 L 10% HCl-washed polycarbonate Nalgene bottles and placed in an outdoor 300 L table receiving high flow-through (100 L min^−1^) water from Old Fort Pond, Southampton, NY, creating temperature and light conditions similar to all NY field sites. For the Lake Erie experiment, bottles were incubated in a wire mesh enclosure and floated in Lake Erie at the Stone Lab of Ohio State University.

**Table 1:** Location, dates, initial cyanobacterial biomass as measured via a bbe Fluoroprobe and parameters measured during eight experiments performed across five locations.

Site	Date	Intial cyanobacterial biomass (μg L^−1^)	Parameters evaluated
Lake Agawam	8/3/21–8/6/21	49	Algal diversity and biomass, microcystin, cyanobacterial diversity, δPO^1^⁵N
Lake Agawam	9/17/23–9/19/23	110	Algal diversity and biomass, microcystin
Mill Pond	7/27/21–7/29/21	18	Algal diversity and biomass, microcystin
Mill Pond	8/10/21–8/13/21	59	Algal diversity and biomass, microcystin, cyanobacterial diversity, δPO^1^⁵N
Mill Pond	8/25/21–8/27/21	72	Algal diversity and biomass
Wainscott Pond	7/14/21–7/16/21	180	Algal diversity and biomass, microcystin, cyanobacterial diversity
Lake in Central Park	7/21/21–7/23/21	130	Algal diversity and biomass
Lake Erie	8/16/21–8/19/21	12	Algal diversity and biomass, cyanobacterial diversity

Initial.

The effects of different nutrient sources were analyzed during experiments. Raw and sand-filtered wastewater was collected from the New York State Center for Clean Water Technology Wastewater Research and Innovation Facility in Stony Brook, NY, United States, that receives wastewater from Suffolk County, NY, pump station #10 prior to sending the wastewater to the Stony Brook University sewage treatment plant. While the ‘raw wastewater’ was collected after settling in a septic tank, the sand-filtered wastewater was collected after the septic tank effluent was passed through a 3 × 3 m cylinder filled with C33 grade sand. These two forms of wastewater represented direct discharge into an ecosystem and discharge after passage through a riparian zone (Freeze and Cherry, 1979), respectively. All wastewater was passed through a sterile, 0.2 μm filter prior to use to remove bacteria and significant amounts of particulate organic carbon. Nutrient solutions were analyzed for total N, total P, total Kjeldahl N (TKN = ammonium plus organic N), nitrate, and orthophosphate on a Lachat Quikchem flow injection system using US EPA methods 351.2, 353.2, 365.1 (US EPA, 1993a, 1993b, 1993c). Raw wastewater contained 3,980 μM N and 221 μM P with all of the N present as ammonium and organic N. The sand filtered wastewater contained 3,780 μM N and 121 μM P with most of the N present as nitrate (80%) and the remainder as ammonium and organic N. There were also differences in the concentrations of chemical oxygen demand (COD) as measured via US EPA method 410.4 (US EPA, 1993d) between wastewater sources. COD is a proxy for dissolved organic carbon in wastewater (Khan et al., 1998; Rieger et al., 2004) and concentrations were 132 ± 49.8 mg L^−1^ in the raw wastewater and 9.9 ± 5.8 mg L^−1^ in the sand filtered wastewater (Table 2). A low phosphorus, ammonium-N commercial fertilizer solution was made to roughly match the concentrations of N and P from the wastewater additions and solutions of ammonium and orthophosphate were made to approximately match the wastewater and fertilizer additions. All nutrient solutions were 0.2 μm filtered and stored frozen as aliquots until use during experiments. The final concentrations of N and P added to experimental vessels were 80 μM N and 4.4 μM P (1.15 mg N L^−1^; 0.13 mg P L^−1^) for raw wastewater, 76 μM N and 3.4 μM P (1.06 mg N L^−1^; 0.10 mg P L^−1^) for sand-filtered wastewater, 74.4 μM N and 5.16 μM P (1.06 mg N L^−1^; 0.16 mg P L^−1^) for the fertilizer, and 75 μM N and 4 μM P (1.05 mg N L^−1^, 0.12 mg P L^−1^) for the inorganic nutrient (ammonium and orthophosphate) treatments (Table 2). N additions were within 6% of each other as there was likely some denitrification within the sand beds (Waugh et al., 2020; Gobler et al., 2021) and P additions were within 25% as the sand filtration binds and removes some P (Wang et al., 2024). After 48 h, bottles were processed for the measurement of phytoplankton pigments, microcystin, δ^15^N content of particulate organic nitrogen (δPO^15^N), and cyanobacterial rRNA (16S SSU) sequencing as described above and below. Differences across the levels of measured parameters were compared by one-way ANOVAs followed by Tukey multiple comparison tests using SigmaPlot v15.

**Table 2:** Concentrations of N and P added to experimental vessels.

Treatment	TN (μM)	TP (μM)	TKN (μM)	NO_3_^−^ (μM)	PO_4_^−3^ (μM)	COD (mg L^−1^)
Inorganic nutrients	75	4.0	75	bdl	4.0	n/m
Fertilizer	74	5.2	74	bdl	5.1	n/m
Sand-filtered wastewater	76	3.4	15	61	2.7	9.9
Raw wastewater	80	4.4	80	bdl	3.3	132

TN and TP are total N and total P whereas TKN is total Kjeldahl N which is comprised of ammonium and organic N. bdl is below detection limit. The limit of detection for nitrate was 0.5 μM.

### Nitrogen isotope analyses

For some experiments, δ^15^N content of particulate organic nitrogen (δPO^15^N) was analyzed on plankton collected on precombusted (4 h at 450°C) glass fiber filters that were dried (24 h @ 60°C), pelleted in tin discs, and analyzed by an Elementar vario Micro Cube elemental analyzer (Elementar Analysensysteme GmbH, Hanau, Germany) interfaced to a PDZ Europa 20–20 isotope ratio mass spectrometer (Sercon Ltd., Cheshire, United Kingdom) at the U.C. Davis Stable Isotope Facility (Davis, CA). Samples were corrected based on batch-specific calibrated reference materials and final δ^15^N values are expressed relative to international air standards for N.

### Cyanobacterial diversity

For some experiments, deoxyribonucleic acid (DNA) samples were obtained on 0.2 μm polycarbonate filters. Samples were frozen in liquid N after collection and then stored in a-80°C freezer prior to DNA extraction. Samples were extracted with a DNeasy® Power Water Kit (Qiagen), modified for cyanobacteria. The quality of DNA was evaluated on a NanoDrop Microvolume Spectrophotometer (ThermoFisher) and the quantity of DNA in extracts was evaluated on a Qubit Fluorometer (ThermoFisher), allowing the concentration of DNA for sequencing to be normalized across all samples. Samples were sent to the Molecular Research Laboratories in Shallowater, TX, for 16S sequencing of 20,000 reads per sample. Sequences were processed using the Quantitative Insights Into Microbial Ecology QIIME 2 (v. 2021.4.0) microbiome analysis software package following the “Moving pictures” pipeline (Bolyen et al., 2019). Briefly, paired-end reads were trimmed of their primers and barcodes using the Cutadapt plugin and then merged by Dada2 to produce a table of exact (100%) amplicon sequence variants (ASV) (Callahan et al., 2016). The sequences clustered into unique ASV’s with 100% sequence identity, with mitochondrial/chloroplast and heterotrophic bacterial ASVs not considered for further analysis. For taxonomic identification of the 16S dataset, the 99% 16S only rep set FASTA and majority consensus seven-level taxonomy files of the SILVA rRNA (16S SSU) release v138 database (Quast et al., 2012) were used to characterize sequences. Samples from sequenced experiments were analyzed microscopically to confirm identifications made via sequencing the 16S rRNA gene.

The relative abundances of cyanobacterial populations from sequences were converted to estimated abundances of cyanobacterial genera following the methods of Ladds et al. (2021). The method specifically applies the relative abundances of cyanobacterial genera identified via sequencing to the total cyanobacterial biomass as determined by the bbe Fluoroprobe to provide estimates of the biomass of individual cyanobacterial genera. While this approach has been used successfully in other cyanobacterial studies (Ladds et al., 2021; Lusty and Gobler, 2023; Kramer et al., 2024), it does have limitations. Cyanobacteria can differ in their pigment content as well as their copy number of the 16S rRNA gene (Klappenbach et al., 2000; Schirrmeister et al., 2012) and this variance could skew the absolute outcome of this approach. Still, given the fluoroprobe data provides data on the biomass of the total cyanobacterial population, the approach here allows for estimates of biomass at the genera level rather than simply the relative abundance data from the 16S rRNA gene sequencing alone. We emphasize this method is one of five approaches used to evaluate experiments (in addition assessments of the absolute fluorometric analyses of phytoplankton groups, the relative abundance of cyanobacteria, microcystin analyses, and δPO^15^N) and that they provide estimates of cyanobacterial biomass at the genus level.

## Results

### Lake Agawam

**Figure 2:**
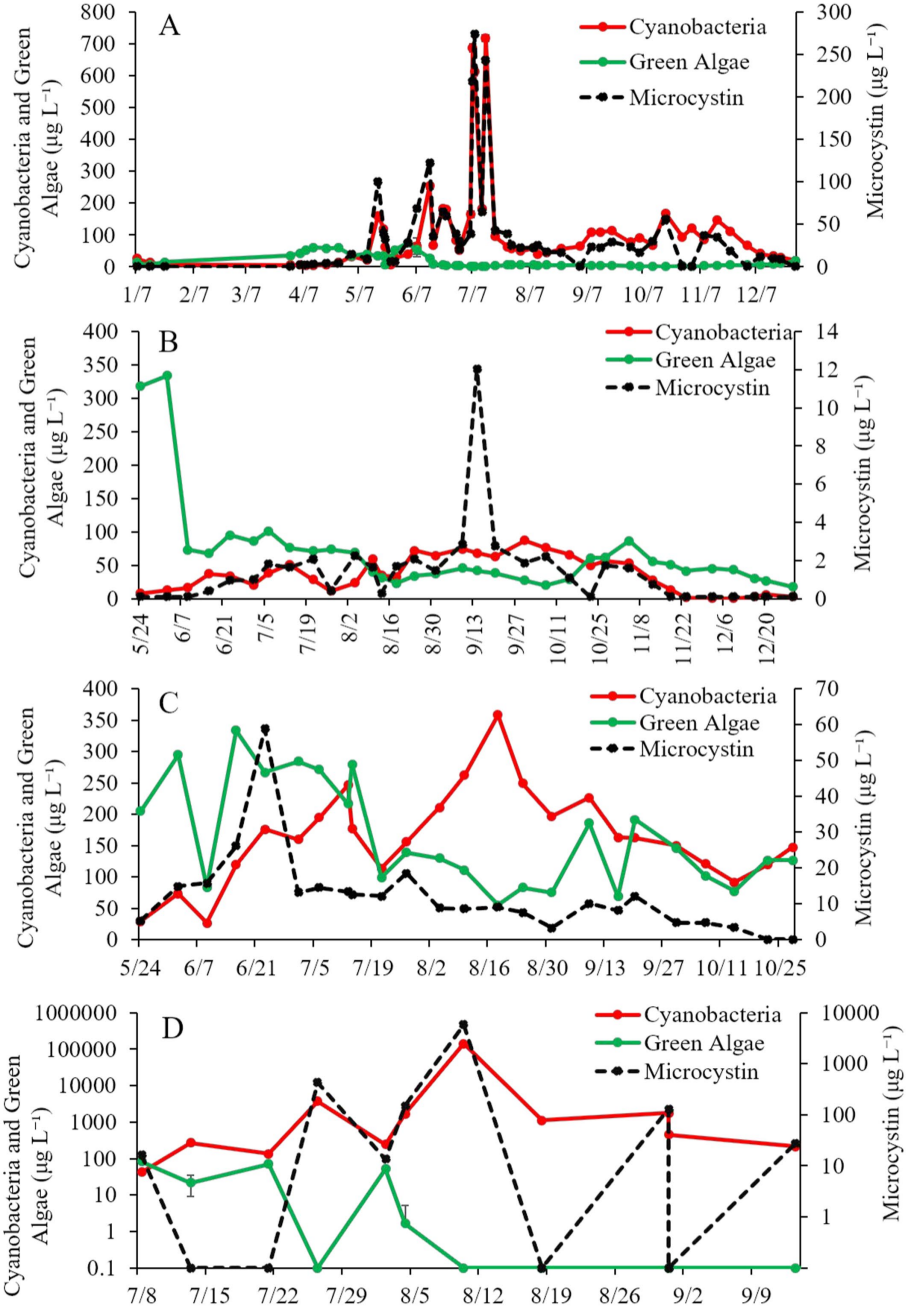
Time-Series of cyanobacteria, green algae, and microcystin concentrations (μg L^−1^) in **(A)** Lake Agawam, **(B)** Mill Pond, **(C)** Wainscott Pond, and **(D)** Lake in Central Park during 2021. Error bars represent +/− standard error.

Cyanobacteria concentrations in Lake Agawam were < 25 μg cyano chl-*α* L^−1^ from January through April in 2021 but were higher May through early December (25.0–719 μg cyano chl-α L^−1^), peaking in mid-July (Figure 2A). Microcystin concentrations followed a similar trend, remaining below 15 μg L^−1^ from January through April before increasing above 15 μg L^−1^ for the remainder of the year, peaking in July at ~27,400 μg L^−1^ (Figure 2A). Green algae concentrations at Lake Agawam ranged undetec_table_ to 60.1 μg green algal chl-α L^−1^ being detectable during winter and spring (Figure 2A).

**Figure 3:**
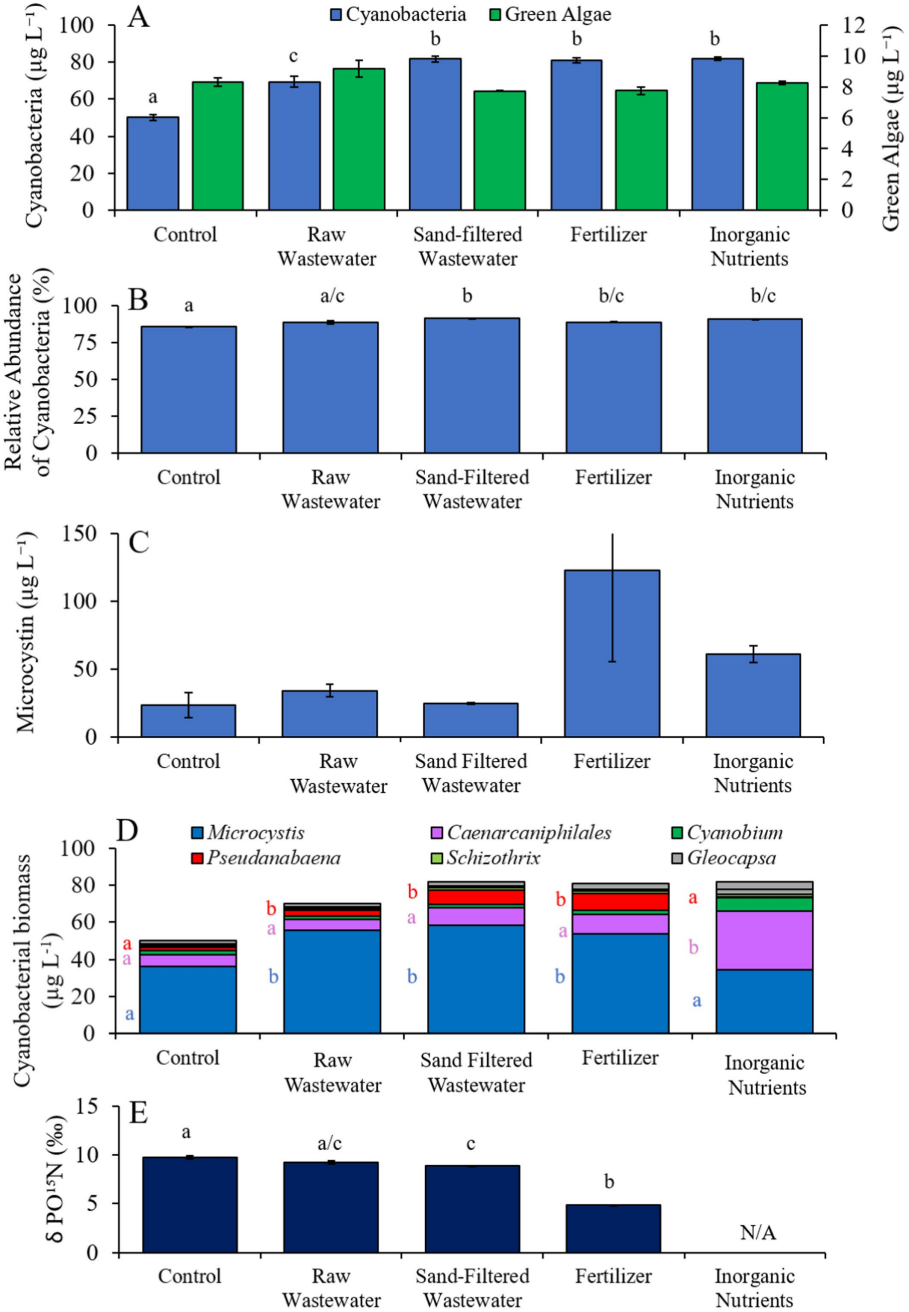
**(A)** Average cyanobacteria and green algae concentration (μg L^−1^), **(B)** relative abundance of cyanobacteria (%), **(C)** average microcystin concentration (μg L^−1^), **(D)** average cyanobacterial biomass (μg L^−1^) separated by species, and (D) average change in δPO^15^N (‰), across different nutrient treatments in Lake Agawam during 8/3/21–8/6/21. Error bars represent +/− standard error. Lowercase letters represent results of pairwise comparisons between groups using Tukey HSD. N/A represents a dataset that was not available.

In the first Lake Agawam experiment (8/3/21–8/6/21), all treatments had higher (Tukey’s HSD; *p* < 0.05) cyanobacteria concentrations (69.4–81.7 cyano chl-*α* L^−1^) compared to the unamended control (50.0 μg cyano chl-*α* L^−1^; Figure 3A), but there was no significant difference in green algae concentration across treatments (Figure 3A). The relative abundance of cyanobacteria was higher (Tukey’s HSD; *p* < 0.05) in the sand-filtered wastewater, fertilizer, and inorganic nutrient treatments, compared to the control (Figure 3B). There were no statistically significant differences in microcystin concentrations across treatments (Figure 3C). Cyanobacterial biomass was lowest in the control and highest in the fertilizer treatment, with *Microcystis*, *Caenarcaniphilales*, and *Pseudanbaena* being the most prominent taxa found across all treatments (Figure 3D). *Microcystis* and *Pseudanbaena* biomass levels were higher (Tukey’s HSD; *p* < 0.05) in the raw wastewater, sand-filtered wastewater and fertilizer treatments, compared to the control and inorganic nutrient treatments, while *Caenarcaniphilales* biomass was higher (Tukey’s HSD; *p* < 0.05) in the inorganic nutrient treatment compared to all other treatments (Figure 3D). δPO^15^N was lower (Tukey’s HSD; *p* < 0.05) in the fertilizer treatment (mean ± S.D. = 4.83 ± 0.04‰) compared to all other treatments (8.87–9.73 ‰) (Figure 3E).

**Figure 4:**
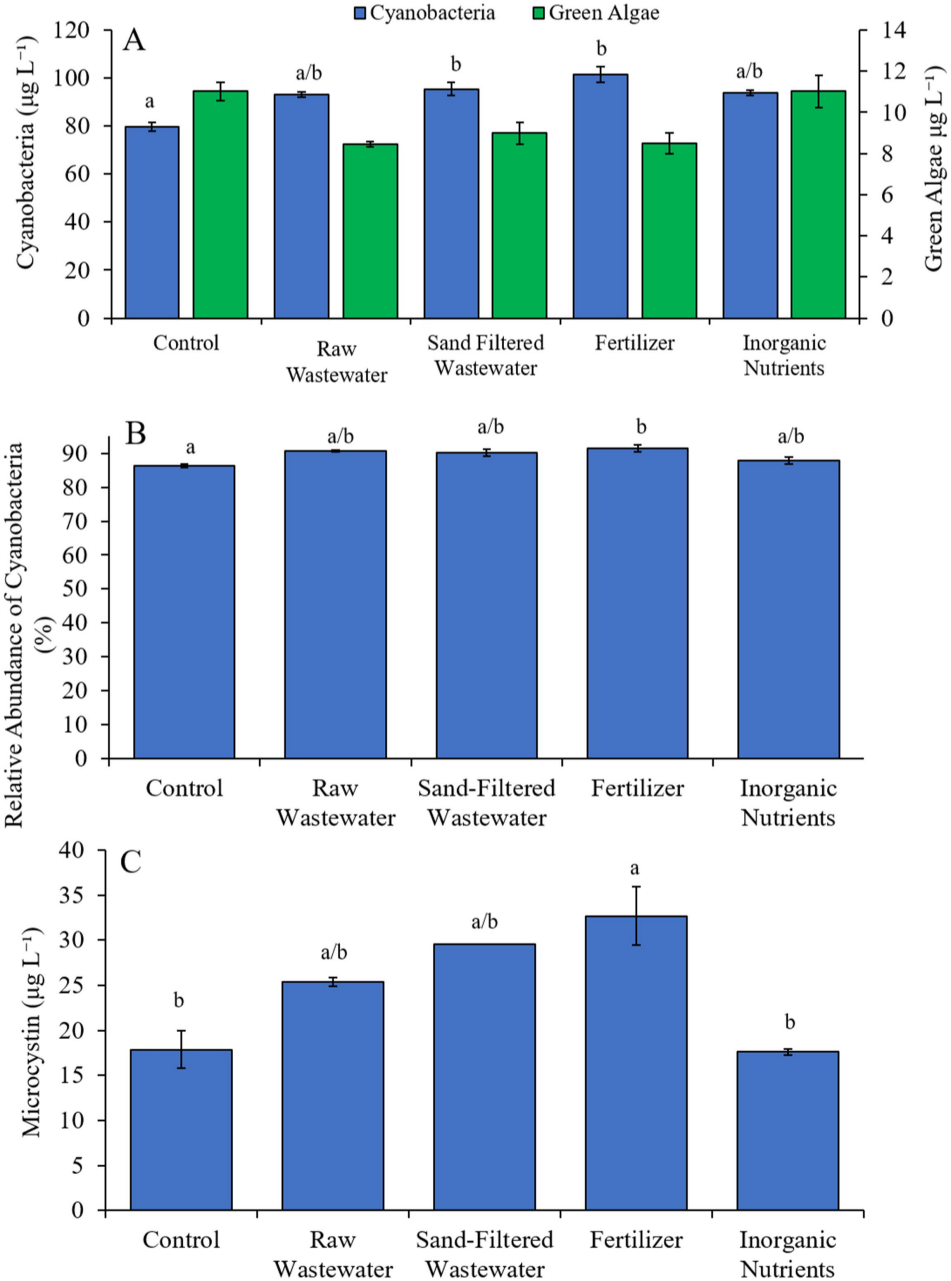
**(A)** Average cyanobacteria and green algae concentration (μg L^−1^), **(B)** relative abundance of cyanobacteria (%), and **(C)** average microcystin concentration (μg L^−1^), across different nutrient treatments in Lake Agawam during 9/17/21–9/19/21. Microcystin concentration was not available for raw wastewater and fertilizer treatments in Lake Agawam during 9/17/21–9/19/21. Error bars represent +/− standard error. Lowercase letters represent results of pairwise comparisons between groups using Tukey HSD. N/A represents a dataset that was not available.

During the second Lake Agawam experiment (9/17/23–9/19/23), cyanobacteria concentrations were higher (Tukey’s HSD; *p* < 0.05) in the sand-filtered wastewater and fertilizer treatments compared to the control (Figure 4A). Green algae concentrations did not differ across treatments (Figure 4A). Only the fertilizer treatment contained a higher (Tukey’s HSD; *p* < 0.05) relative abundance of cyanobacteria compared to the control (Figure 4B). Similarly, microcystin concentrations were higher (Tukey’s HSD; p < 0.05) only in the fertilizer (32.7 ± 3.24 μg L^−1^) treatment compared to the control (17.9 ± 2.11 μg L^−1^) (Figure 4C).

### Mill Pond

Cyanobacteria concentrations in Mill Pond ranged ~8–16 μg cyano chl-*α* L^−1^ in late May and early June, increased from mid-June through mid-November (~20 to ~72 μg cyano chl-α L^−1^) and then declined below ~6 μg cyano chl-α L^−1^ through the rest of 2021 (Figure 2B). In contrast, green algae concentrations at Mill Pond peaked in early June-2021 at ~334 μg green algal chl-α L^−1^ and ranged from ~17–101 μg green algal chl-α L^−1^ thereafter (Figure 2B). Microcystin concentrations ranged below detection to ~3 μg L^−1^ from May through December at Mill Pond, apart from 9/14/21 when concentrations reached ~12 μg L^−1^ (Figure 2B).

**Figure 5:**
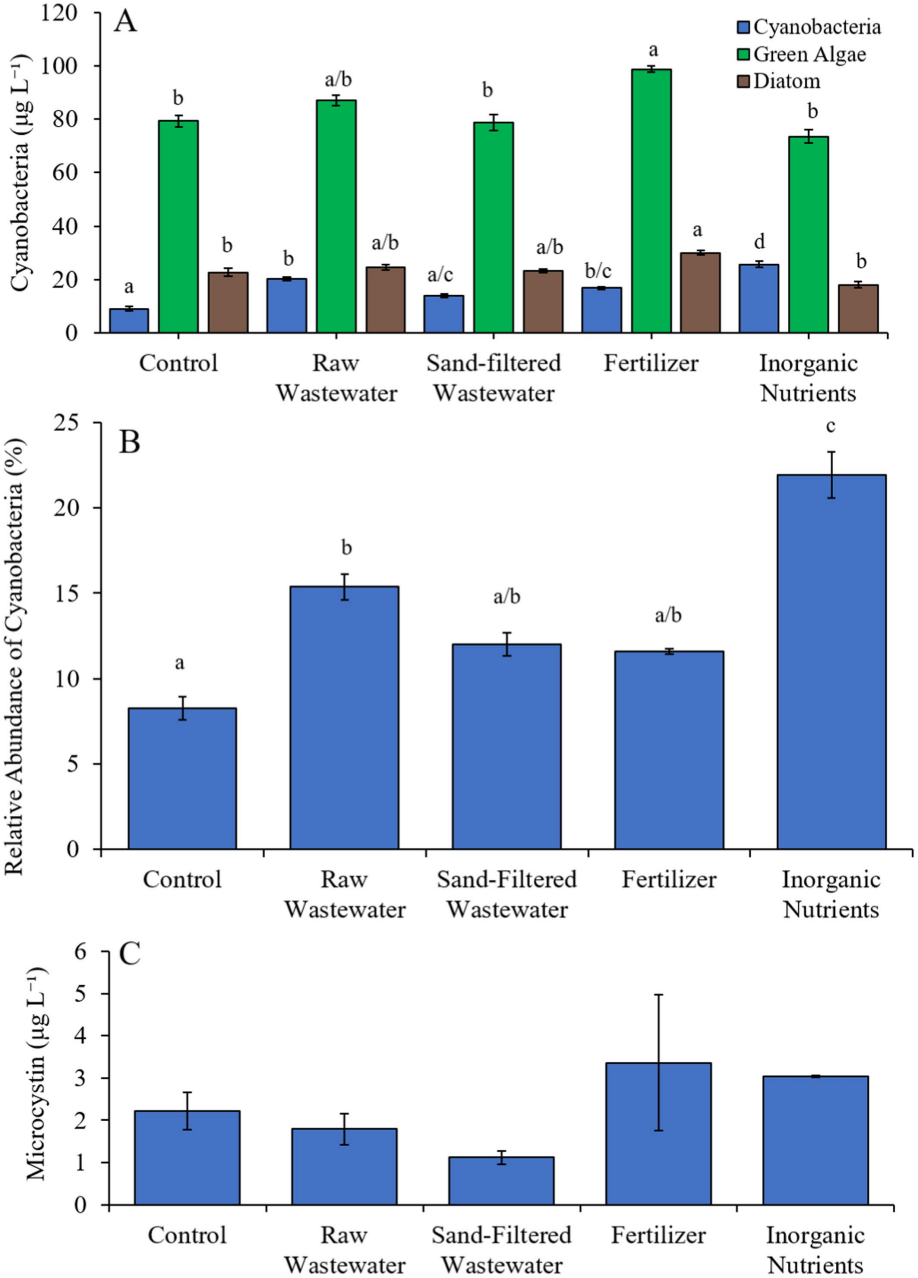
**(A)** Average cyanobacteria, green algae, and diatom concentration (μg L^−1^), **(B)** relative abundance of cyanobacteria (%), and **(C)** average microcystin concentration (μg L^−1^) across different nutrient treatments in Mill Pond during 7/27/21–7/29/21.

During the first Mill Pond experiment (7/27/21–7/29/21), cyanobacterial concentrations were higher (Tukey’s HSD; *p* < 0.05) in the raw wastewater (20.29 μg cyano chl-*α* L^−1^), fertilizer (16.9 μg cyano chl-α L^−1^) and inorganic nutrient (25.8 μg cyano chl-α L^−1^) treatments compared to the control (9.18 μg cyano chl-α L^−1^) (Figure 5A). Green algae concentrations were higher (Tukey’s HSD; *p* < 0.05) in the fertilizer treatment than in the control only (Figure 5A). Similarly, diatom concentrations were higher (Tukey’s HSD; *p* < 0.05) only in the fertilizer treatment compared to the control (Figure 5A). The relative abundance of cyanobacteria was higher (Tukey’s HSD; *p* < 0.05) in the raw wastewater (15.4%) and inorganic nutrient (22.0%) treatments, compared to the control (8.26%) (Figure 5B). The inorganic nutrient treatment contained a higher relative abundance of cyanobacteria compared to all treatments (Tukey’s HSD; *p* < 0.05; Figure 4B). There was no difference in microcystin concentrations across treatments (Figure 5C).

**Figure 6:**
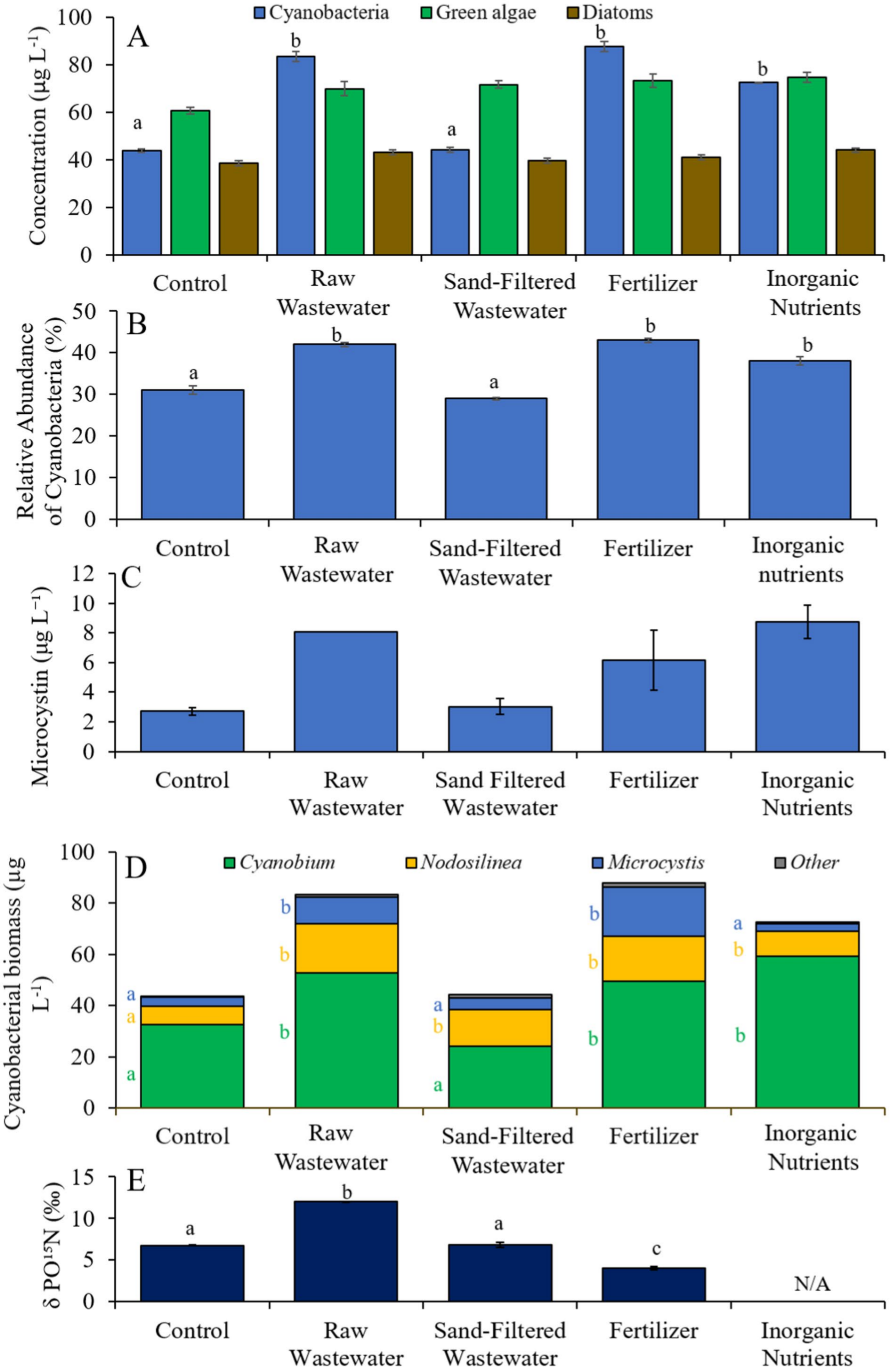
**(A)** Average cyanobacteria, green algae, and diatom concentration (μg L^−1^), **(B)** relative abundance of cyanobacteria (%), **(C)** average microcystin concentration (μg L^−1^), **(D)** average cyanobacterial biomass (μg L^−1^) separated by species, and **(E)** average change in δPO^15^N (‰), across different nutrient treatments in Mill Pond during 8/10/21–8/13/21. Error bars represent +/− standard error. Lowercase letters represent results of pairwise comparisons between groups using Tukey HSD. N/A represents a dataset that was not available.

During the second Mill Pond experiment (8/10/21–8/13/21), cyanobacterial concentrations and the relative abundances of cyanobacteria were higher (Tukey’s HSD; *p* < 0.05) in the fertilizer, raw wastewater, and inorganic treatments than in the control (Figures 6A,B). There was no difference in green algae, diatom, or microcystin concentrations between treatments (Figures 6A,C). Cyanobacterial biomass was lowest in the control and highest in the fertilizer treatment, with *Microcystis*, *Cyanobium*, and *Nodosilinea*, being the most prominent genera found across all treatments (Figure 6D). *Microcystis* levels were higher (Tukey’s HSD; *p* < 0.05) in the raw wastewater and fertilizer treatments than in the control (Figure 6D). *Cyanobium* concentrations were higher (Tukey’s HSD; *p* < 0.05) in the raw wastewater, fertilizer, and inorganic nutrient treatments compared to the control treatment, while *Nodosiliniea* concentrations were higher (Tukey’s HSD; *p* < 0.05) in all treatments compared to the control (Figure 6D). δPO^15^N was higher (Tukey’s HSD; *p* < 0.05) in the raw wastewater (12.1‰) treatment compared to all other treatments and lower (Tukey’s HSD; *p* < 0.05) in the fertilizer (4.02‰) treatment compared to all other treatments (Figure 6E).

**Figure 7:**
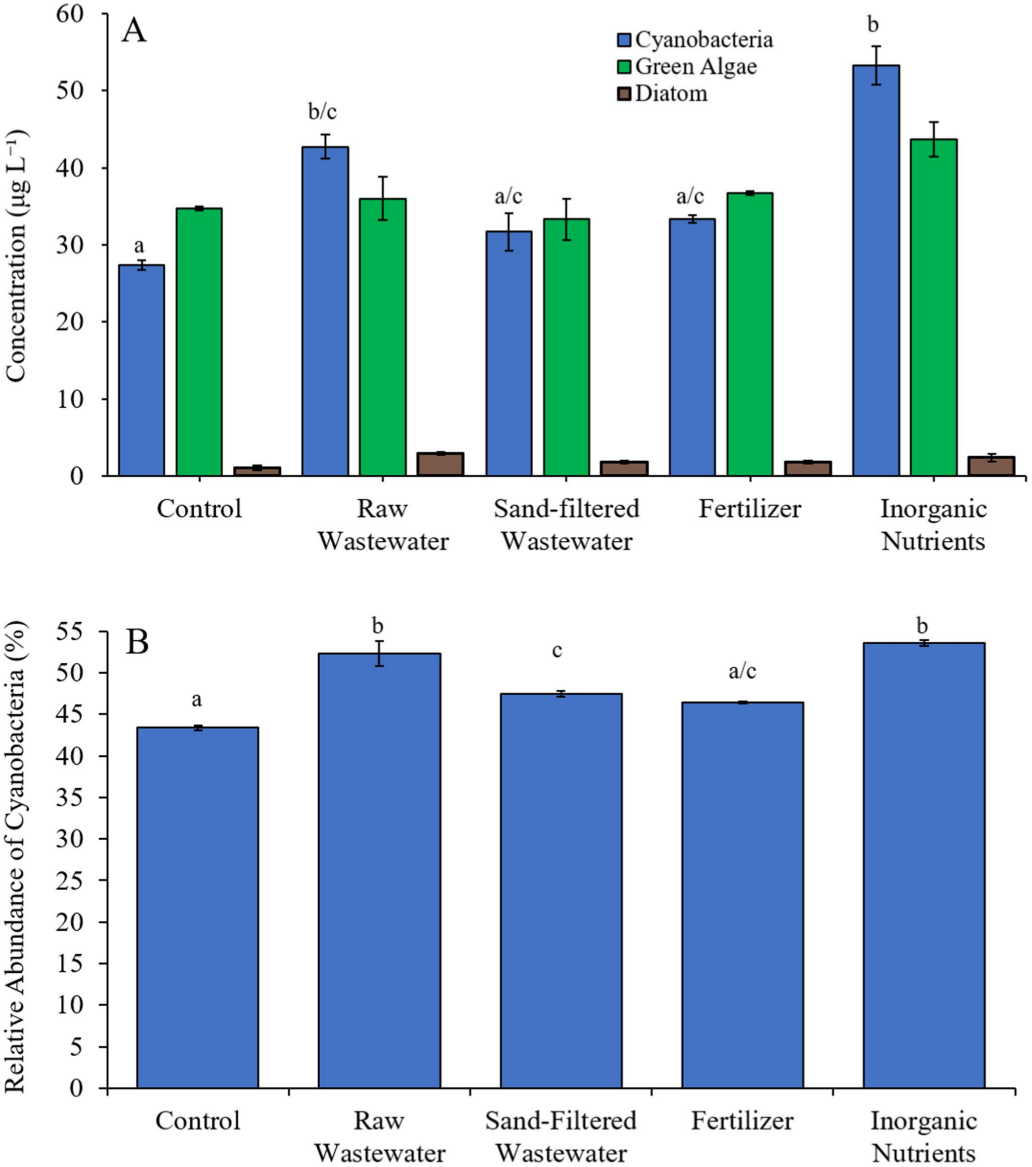
**(A)** Average cyanobacteria, green algae, and diatom concentration (μg L^−1^), and **(B)** relative abundance of cyanobacteria (%), across different nutrient treatments in Mill Pond during 8/25/21–8/27/21. Error bars represent +/− standard error. Lowercase letters represent results of pairwise comparisons between groups using Tukey HSD.

During the third Mill Pond experiment (8/25/21–8/27/21), cyanobacterial concentrations were higher (Tukey’s HSD; *p* < 0.05) in the raw wastewater (42.8 μg cyano chl-*α* L^−1^) and inorganic nutrient (53.3 μg cyano chl-α L^−1^) treatments compared to the control (27.4 μg cyano chl-α L^−1^) (Figure 7A). However, there was no significant difference in green algae nor diatom concentrations between any treatment (Figure 7A). The relative abundance of cyanobacteria in the raw wastewater (52.3%), sand-filtered wastewater (47.5%) and inorganic nutrient (53.6%) treatments were higher than in the control (43.4%; Tukey’s HSD; *p* < 0.05; Figure 7B).

### Wainscott Pond

Cyanobacteria concentrations in Wainscott Pond ranged ~29–358 μg cyano chl-*α* L^−1^ in 2021, peaking in mid-August (Figure 2C). Green algae concentrations ranged ~55–333 μg green algal chl-α L^−1^ throughout the year, peaking in mid-June (Figure 2C). Microcystin concentrations ranged ~3 to ~59 μg L^−1^ and peaked in late June (Figure 2C).

**Figure 8:**
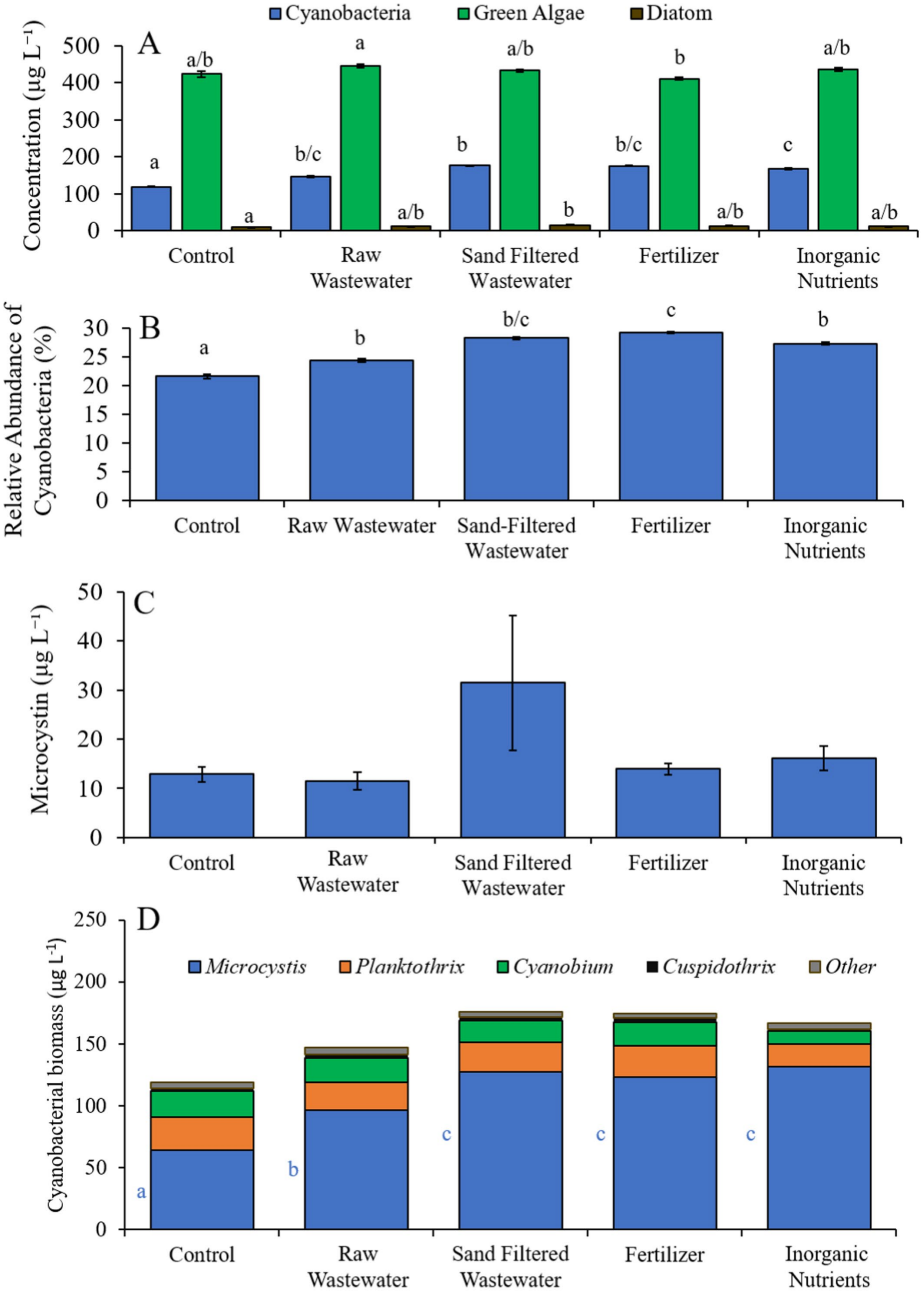
**(A)** Average cyanobacteria, green algae, and diatom concentration (μg L^−1^), **(B)** relative abundance of cyanobacteria (%), **(C)** average microcystin concentration (μg L^−1^), and **(D)** average cyanobacterial biomass (μg L^−1^) separated by species, across different nutrient treatments in Wainscott Pond during 7/14/21–7/16/21. Error bars represent +/− standard error. Lowercase letters represent results of pairwise comparisons between groups using Tukey HSD.

During the Wainscott Pond experiment (7/14/21–7/16/21), cyanobacterial concentrations were higher (Tukey’s HSD; *p* < 0.05) in all treatments (146–176 μg cyano chl-*α* L^−1^) compared to the control (110 μg cyano chl-α L^−1^; Figure 8A). Green algae concentrations across treatments were not different from the control. Diatom concentrations were higher (Tukey’s HSD; *p* < 0.05) only in the sand-filtered wastewater (15.2 μg diatom chl-α L^−1^) treatment compared to the control (8.65 μg diatom chl-α L^−1^) (Figure 8A). All treatments contained a higher (Tukey’s HSD; *p* < 0.05) relative abundance of cyanobacteria (24.3–29.2%) than the control (21.6%) (Figure 8B). The fertilizer (29.2%) treatment also had a higher (Tukey’s HSD; *p* < 0.05) relative abundance of cyanobacteria compared to all treatments (Figure 8B). There were no differences in microcystin concentration across treatments (Figure 8C). Cyanobacterial biomass was lowest in the control and highest in the fertilizer treatment, with *Cuspidothrix, Cyanobium, Planktothrix,* and *Microcystis* being the most prominent genera (Figure 8D). While *Cuspidothrix, Cyanobium,* and *Planktothrix* levels were not different between treatments, *Microcystis* concentrations were higher (Tukey’s HSD; p < 0.05) in all treatments compared to the control (Figure 8D).

### Lake in Central Park

During the summer of 2021, cyanobacteria concentrations at the Lake in Central Park averaged ~13,900 μg cyano chl-α L^−1^ and reached ~29,900 μg cyano chl-α L^−1^ on 21-July-2021 and ~ 140,000 μg cyano chl-α L^−1^ on 10-August-2021 (Figure 2D). Green algae concentrations ranged ~0–82 μg green algal chl-α L^−1^, peaking in early July, while microcystin concentrations ranged ~16–5,860 μg L^−1^, with the peak occurring in mid-August (Figure 2D).

**Figure 9:**
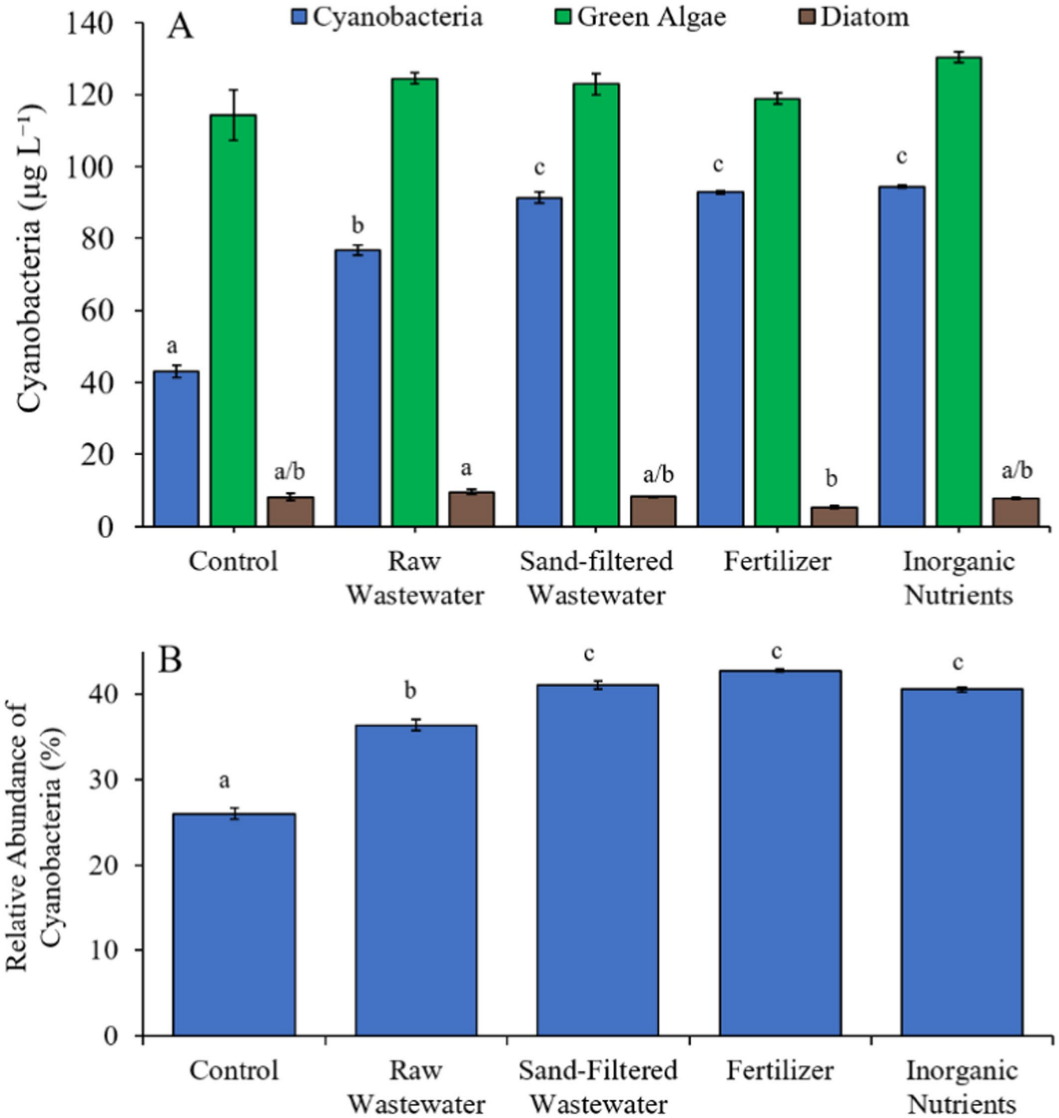
**(A)** Average cyanobacteria and green algae concentration (μg L^−1^), and **(B)** relative abundance of cyanobacteria (%) across different nutrient treatments (after 48 h) in Central Park Lake during 7/21/21–7/23/21. Error bars represent +/− standard error. Letters represent results of pairwise comparisons between groups using Tukey HSD.

During the Lake in Central Park (7/21/21–7/23/21) experiment, cyanobacterial concentrations were higher (Tukey’s HSD; *p* < 0.05) in all treatments (76.8–94.4 μg cyano chl-α L^−1^) compared to the control (43.1 μg cyano chl-α L^−1^) (Figure 9A). There was no difference in green algae nor diatom concentration between treatments (Figure 9A). All treatments contained a higher (Tukey’s HSD; *p* < 0.05) relative abundance of cyanobacteria (36.4–42.8%) compared to the control (26.0%) (Figure 9B).

### Lake Erie

**Figure 10:**
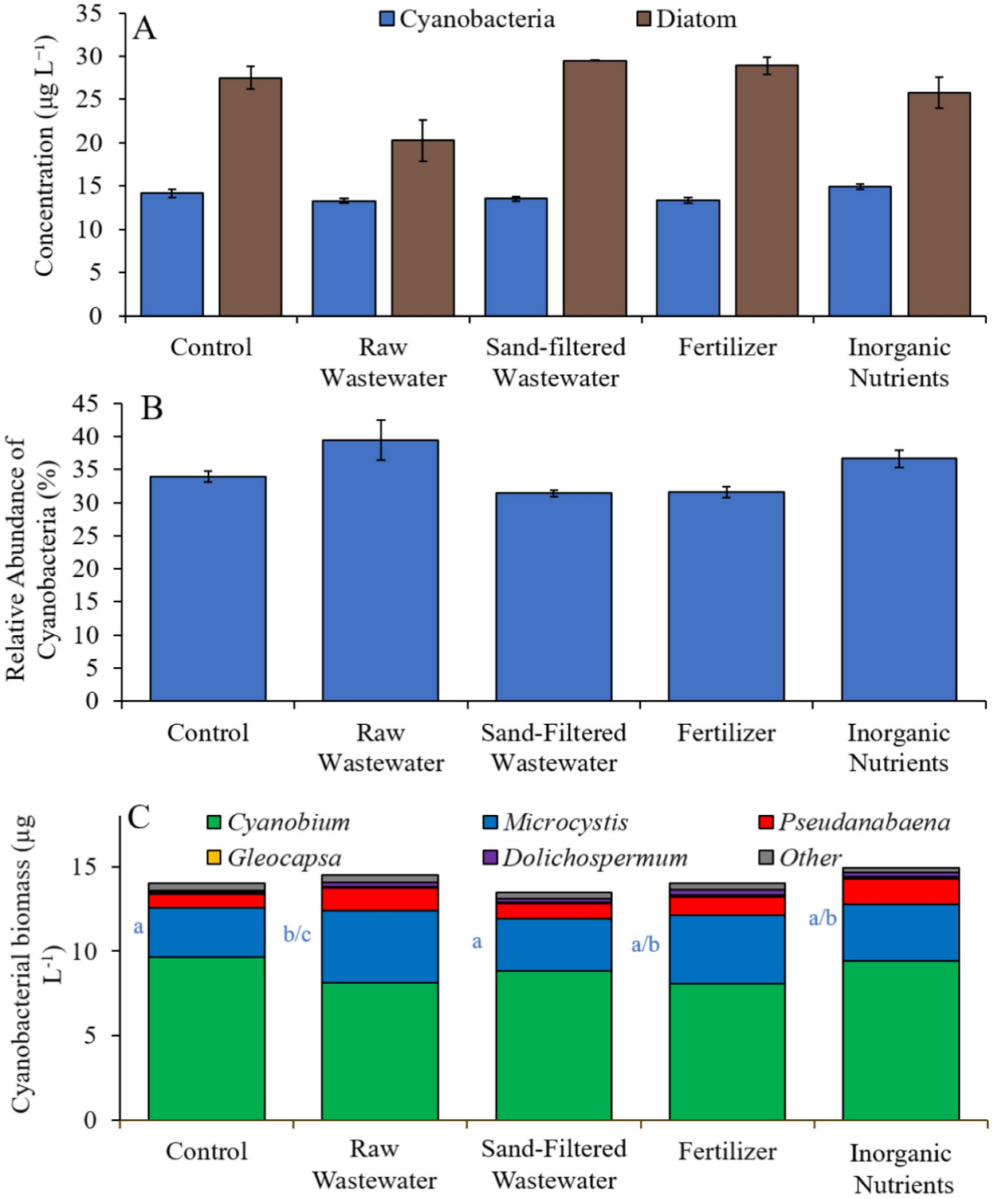
**(A)** Average cyanobacteria and green algae concentration (μg L^−1^), **(B)** relative abundance of cyanobacteria (%), and **(C)** average cyanobacterial biomass (μg L^−1^) separated by species, across different nutrient treatments in Lake Erie during 8/16/21–8/19/21. Error bars represent +/− standard error.

Finally, during the Lake Erie experiment (8/16/21–8/19/21), cyanobacteria concentrations, cyanobacterial relative abundance, green algae concentrations, and diatom concentrations did not differ between treatments (Figures 10A,B). Cyanobacterial biomass was lowest in the sand-filtered wastewater treatment (14.2 μg cyano chl-α L^−1^) and highest in the inorganic nutrient (17.1 μg cyano chl-α L^−1^) treatment, with *Cyanobium* and *Microcystis* being the most prominent taxa found across the treatments (Figure 10C). While *Cyanobium* concentrations were not different between treatments (Figure 10C), *Microcystis* concentrations were higher (Tukey’s HSD; *p* < 0.05) in the raw wastewater (4.85 μg cyano chl-α L^−1^) treatment compared to the control (3.15 μg cyano chl-α L^−1^; Figure 10C).

## Discussion

During this study, wastewater and fertilizer-derived nutrients increased cyanobacterial biomass, and in some cases, microcystin levels, while rarely doing so for other, co-occurring groups of phytoplankton (green algae, diatoms). A combined gene sequencing-fluorometric approach identified multiple genera of bloom-forming cyanobacteria that increased in abundance in response to different nutrient sources with *Microcystis* increasing in biomass in response to raw wastewater in every experiment in which genus-specific responses were evaluated. Wastewater and fertilizer increased and decreased δPO^15^N values, respectively, revealing the potential for δPO^15^N to be used as a diagnostic for the source of nutrients utilized by cyanobacteria-dominated plankton communities. Collectively, these findings bring new insight into the role fertilizer-and wastewater-derived nutrients play in promoting CHABs.

CHABs are promoted by excessive loading of N and P to freshwater ecosystems (Paerl et al., 2011, 2016; Paerl, 2018). During this study, responses of CHABs to differing nutrient sources with near-equimolar N and similar P levels were often highly similar. Fertilizer, inorganic nutrients, raw wastewater, and sand-filtered wastewater increased cyanobacterial biomass and the relative abundance of cyanobacteria in the large majority of experiments. In contrast, the responses of green algae and diatoms were highly muted, with green algae experiencing increased biomass in only one treatment during one experiment and diatoms experiencing increased biomass in single treatments during two experiments, potentially due to the ability of diatoms to intracellularly store nitrate (Stief et al., 2022). Collectively this demonstrates that excessive anthropogenic nutrients loading can selectively promote CHABs to the exclusion of other phytoplankton.

Globally, fertilizer is the largest source of nutrients from land to coastal water bodies (Canfield et al., 2010; Fink et al., 2018) but in more urbanized watersheds, wastewater is typically the dominant source of nutrients (Castro et al., 2003; Latimer and Charpentier, 2010; Jenny et al., 2016). Given the similarity of responses across nutrient sources, this study has demonstrated the near equal ability of wastewater and fertilizer to promote CHABs. While all nutrient solutions used during this study contained N and P, the fertilizer solution also contained potassium, although this was unlikely to strongly alter microbial growth given its already high concentrations in freshwater systems (Brakke et al., 1988). In contrast, wastewater contains a myriad of elements (Henze and Comeau, 2008) that could have altered the growth and abundance of CHABs. Given all wastewater solutions were sterile-filtered prior to use, they did not contain bacteria but did contain viruses, most of which were unlikely to be specific for surface water microbes (Weitz and Wilhelm, 2012). Wastewater solutions, particularly raw wastewater, were also enriched in organic carbon that may have been an important nutrient for cyanobacteria and/or heterotrophic bacteria (Paerl, 1977; Harke and Gobler, 2013; Zhao et al., 2019). Given that recent studies have identified the critical role that endosymbiotic bacteria within *Microcystis* colonies can play in the physiology of these CHABs (Jankowiak and Gobler, 2020; Cook et al., 2020; Hoke et al., 2021), stimulation of these heterotrophic bacteria via wastewater could indirectly promote *Microcystis*. Finally, wastewater was likely enriched in trace elements such as iron that may have altered microbial growth (North et al., 2007). Still, given that the responses of cyanobacteria to inorganic nutrients, fertilizer, raw wastewater, and sand-filtered wastewater were broadly similar, it would seem the strongest driver across experiments was N and *P. prior* studies of several of these systems (Lake Erie, Lake Agawam, Lake in Central Park) have identified N as limiting the indigenous CHABs (Flanzenbaum et al., 2022; Gobler et al., 2007; Davis et al., 2010; Chaffin et al., 2013), suggesting that N was most important for the growth responses of cyanobacteria to nutrient solutions during this study.

Among the nutrient treatments, sand-filtered wastewater less consistently increased cyanobacterial biomass and microcystin than others. Fertilizer, inorganic nutrients, raw wastewater, significantly increased cyanobacterial biomass in 75% of experiments while and sand-filtered wastewater did so in only 50% of experiments and sand-filtered nutrients never caused an increase in microcystin. The sand-filtered wastewater treatment was meant to mimic flow of water through a riparian zone, prior to surface water discharge (Freeze and Cherry, 1979). In addition, sand beds are a common wastewater treatment approach that can remove the biological oxygen demand, orthophosphate, and organic carbon, while concurrently promoting nitrification of ammonium (Rodgers et al., 2005; Gobler et al., 2021). Consistent with these processes, sand filtered-wastewater contained 25% lower levels of P, 90% lower levels of ammonium and organic nitrogen (TKN), 90% lower levels of COD, and 75% higher levels of nitrate compared to raw wastewater. The lower levels of P and the abundance of nitrate rather than ammonium could both have been responsible for the lowered ability of sand-filtered wastewater to promote CHABs in experiments as consistently as other nutrient sources (Smith, 1979; Schindler, 1977; McCarthy et al., 2009; Glibert et al., 2015). Emerging research has demonstrated that nitrification of ammonium can be a process that the makes CHABs less competitive in Lake Erie where blooms have been associated with the use of ammonium over nitrate (Hampel et al., 2019) and nitrification rates have been shown to be negatively correlated with cyanobacterial biomass (Hoffman et al., 2023). These findings also point to a potential means for the mitigation of CHABs in regions with limited means for sewage treatment, specifically demonstrating that the discharge of raw wastewater through a sand-bed can lessen the likelihood of promoting these events compared to direct discharge by removing organic matter and P and converting ammonium to nitrate via nitrification. Coupling nitrifying sand-beds with a denitrification step to remove N from wastewater, for example via lignocellulose beds (Gobler et al., 2021), would likely be even more effective means of CHAB mitigation (Gobler et al., 2016).

Historically, ^15^N signatures have been used to identify sources of nitrogen in water and plankton with lighter, negative values being reflective of fertilizer-derived N and heavier values greater than 6–10 being indicative of wastewater derived-N (McClelland and Valiela, 1998; Fry, 2006; Bannon and Roman, 2008; Jankowski et al., 2012). During this study, wastewater and fertilizer were capable of increasing and decreasing the δPO^15^N, respectively, outcomes consistent with the known δ^15^N signatures of these N sources (McClelland and Valiela, 1998; Fry, 2006; Bannon and Roman, 2008; Jankowski et al., 2012). One factor complicating the use of δ^15^N signatures to trace nutrient sources during CHABs is the ability of some cyanobacteria to fix nitrogen, a process that selectively incorporates lighter N and, therefore, lowers ^15^N signatures (Fry, 2006; Jankowski et al., 2012). Hence, in communities dominated by diazotrophic genera, assimilation of fertilizer-derived N could have no impact on an already light δPO^15^N. Conversely, in cases such as in Lake Agawam where δ^15^N signatures were already heavy (~10 ppt), the addition of wastewater had no effect on δ^15^N signatures whereas fertilizers did. Lake Agawam is surrounded by the Village of Southampton, a municipality where wastewater is discharged to ground, mixes with groundwater, and seeps into the lake, suggesting that the heavy δ^15^N signatures there were caused by usage of wastewater-derived N by the N-limited CHABs that have occurred there for decades (Gobler et al., 2007; Gobler et al., 2021). Collectively, these findings reveal the potential for and, limitation of, using δPO^15^N as a diagnostic tool to trace the source of nitrogen utilized by cyanobacteria-dominated plankton communities.

Using the combined high throughput gene sequencing—fluorometric approach (Ladds et al., 2021), multiple genera of cyanobacteria were found to have experienced increased biomass during experiments including *Microcystis, Pseudanabaena, Nodosilinea,* and *Cyanobium*. Among these genera, *Microcystis* was the genus that was most consistently promoted by nutrients, experiencing increased biomass in all four experiments where high throughput gene-sequencing was performed. And while *Microcystis* biomass levels increased in all different treatments across all experiments, raw wastewater was the only treatment to increase *Microcystis* biomass in all four experiments evaluated. As described above, raw wastewater was enriched in ammonium, organic nitrogen, and organic matter, nutritional factors known to promote the growth of *Microcystis* (Berman and Chava, 1999; Harke and Gobler, 2013; Gobler et al., 2016; Krausfeldt et al., 2019). For example, *Microcystis* blooms in western Lake Erie have been linked to a seasonal decline in nitrate and an increase in the loading of reduced species of N (Chaffin et al., 2013; Newell et al., 2019) and the seasonal shifts in N availability can alter the prevalence of differing microcystin congeners of differing toxicity (Chaffin et al., 2023). *Microcystis* blooms are also known to be supported by urea which would be enriched in raw wastewater (Krausfeldt et al., 2019). Elevated levels of organic matter in raw wastewater are also likely to promote the growth of heterotrophic bacteria (Paerl, 1977; Kirchman, 2010) and recent studies have emphasized the role of heterotrophic bacteria within the phycosphere of *Microcystis* colonies in promoting the growth of this CHAB (Jankowiak and Gobler, 2020; Cook et al., 2020; Smith et al., 2021; Hoke et al., 2021). Hence, the increased growth of *Microcystis* could have been due to direct use of nutrients from the wastewater and/or enhanced growth of bacteria that were beneficial to *Microcystis*.

## Conclusion

Nutrients are known to promote CHABs and fertilizer and wastewater are the two most prominent sources of anthropogenic nutrients to surface waters. Here, experiments demonstrated that fertilizer and wastewater can intensify CHAB biomass, relative abundance, and toxicity, with *Microcystis* benefiting from raw wastewater more commonly than other genera and more commonly than from other nutrient sources. These findings emphasize the crucial role of reducing N and P loading for mitigating CHABs (Paerl et al., 2011, 2016; Paerl, 2018). The ability of sand filtering to remove organic carbon and P and to promote nitrification, converting ammonium to nitrate, contributed toward a weaker response in this treatment from cyanobacteria comparted to raw wastewater and demonstrates it efficacy as a simple nutrient mitigation approach to partly mitigate CHABs.

## Data Availability

The original contributions presented in the study are publicly available. This data can be found here: https://www.ncbi.nlm.nih.gov/, BioProject ID PRJNA1178324.
